# Ultrasound-Guided Long Peripheral Catheters for Venous Access in the Intensive Care Unit: A Descriptive Study

**DOI:** 10.7759/cureus.77474

**Published:** 2025-01-15

**Authors:** Amal F Sam, Sruthi Shankar, K Nandakumar, Atchya A Kumar

**Affiliations:** 1 Anaesthesiology, Dr. Rela Institute and Medical Centre, Chennai, IND; 2 Gastrointestinal and Renal Critical Care, Apollo Hospitals, Chennai, IND

**Keywords:** deep arm vein catheters, leadercath, long peripheral catheters, long peripheral venous catheters, sonographically inserted peripheral vein catheters, ultrasound-guided peripheral venous access

## Abstract

Background: Central venous catheters (CVCs) are the choice of venous access if patients require vasoactive drugs or multiple infusions but are not free of adverse effects. They should be removed when deemed unnecessary. In cases of difficult venous access, long peripheral catheters (LPCs) placed in the bigger veins of the arm under sonographic guidance are a good option. Therefore, we conducted this study to evaluate their usefulness.

Methods: This prospective descriptive study evaluated LPCs for venous access placed under ultrasound guidance in one of the three major veins of the upper extremity, namely the basilic, brachial, and cephalic veins. For this purpose, we used the LeaderCath™ (Vygon, Paris, FRA).

Results: This descriptive study included 38 patients with LPCs. Difficult peripheral venous access was the most common indication for LPC placement (44.7%). The second most common reason was removing old CVCs from patients requiring frequent sampling (31.6%). The median duration of the procedure was three minutes (IQR: 1.5 to 10 minutes). The median dwell time for LPC was eight days (IQR: five to 11 days, maximum 30 days). Twenty-six (68.4%) patients had good backflow until removal, which served as a channel for aspirating blood for sampling. Patients with LPCs had better comfort scores than those with CVC (8 (6, 8) vs. 5 (5, 6); p = 0.008).

Conclusion: To secure peripheral venous access under sonographic guidance, LPCs would provide additional advantages such as a channel for backflow and sampling and longer dwell time compared to conventional peripheral intravenous cannulas.

## Introduction

Many patients in our intensive care unit (ICU) require central venous catheters (CVCs) for special drugs, vasoactive drugs, and ease of multiple infusions. Some patients require repeated venous blood sampling during their course in the ICU, and CVC has the additional advantage of acting as a channel to aspirate blood. The CVC placement involves the risk of pneumothorax and hematoma, and its long-term use is associated with an increased risk of infection and central vein stenosis [1-3]. Peripheral vein catheters (PVCs) do not serve the purpose of a channel for aspirating blood; in patients with longer ICU stays, there can be issues such as difficult access with or without peripheral edema [4]. If venous access is placed in larger veins, such as the basilic, cephalic, and brachial veins of the arm, drug infusions and fluids can be administered, and such venous access may also serve as a channel for venous blood aspiration for sampling. This type of access is preferred with a long peripheral catheter (LPC), as per Qin et al., which is usually inserted high in the forearm, cubital fossa, or arm and does not reach the axillary vein [5]. An LPC may alleviate the need for CVC placement in clinically stable patients and, hence, avoid complications such as pneumothorax and hematoma in the neck. The purpose of this study is to analyze the ease of insertion and comfort of care of LPC using the LeaderCath^TM^ (Vygon, Paris, FRA), which is conventionally used as an arterial catheter.

## Materials and methods

This prospective descriptive study was conducted in the liver ICU of Apollo Hospitals, Chennai, from the 17th of October 2022 to the 18th of April 2023. We evaluated the performance of an LPC placed under ultrasound guidance using the Seldinger technique. After obtaining ethical committee approval from Apollo Hospitals, Chennai, TN, IND (approval no: AMH-C-S-042/08-22) and informed consent from participants, we included consecutive patients aged > 18 years who required at least 12-hourly (twice a day) blood samples with an anticipated dwell time of > 72 hours. Patients with CVC showing clinical improvement and awaiting removal of the CVC or clinically stable patients with difficult venous access awaiting peripheral venous catheter insertion were screened. If their frequency of sampling was more than twice a day, they were included in the study and were scheduled to undergo LPC placement.

Patients who refused to participate and patients with infection at the insertion site, thrombus in the vein, or chronic kidney disease were also excluded. Patients who did not show clinical improvement and those with hemodynamic instability who may receive fluid boluses and/or vasopressors were not considered for LPC as it is not ideal for rapid fluid boluses. When no vein other than the vein of interest is seen as patent sonographically at the level of insertion, the patient was excluded from the study. As this is a descriptive study, we did not calculate the required sample size and conducted the study for six months.

**Figure 1:**
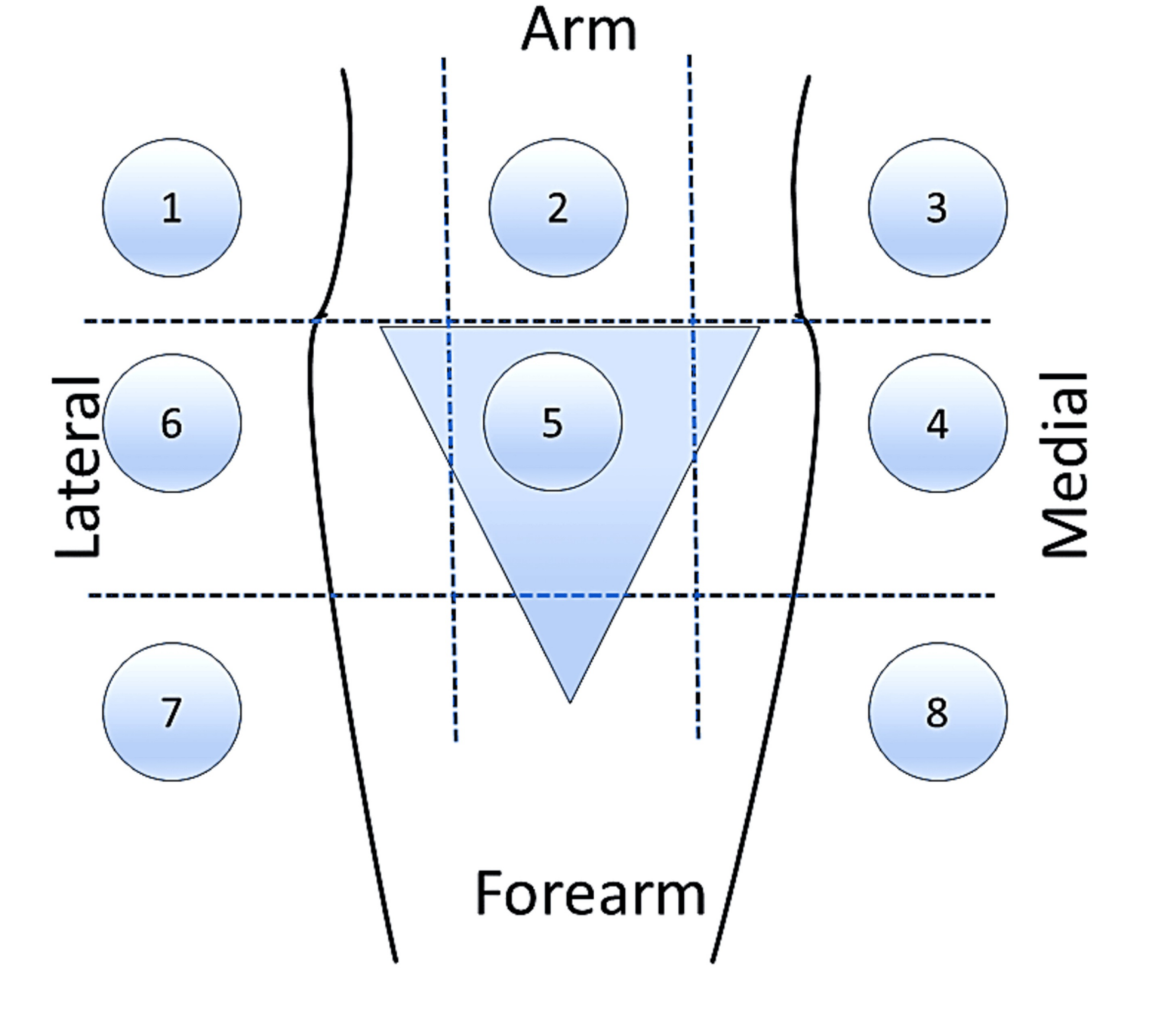
Diagram showing eight available quadrants for catheter placement Upper territory: The arm and cubital fossa (blue triangle); Lower territory: The forearm; Medial territory: Basilic vein; Middle territory: Brachial vein; Lateral territory: Cephalic vein

The recruited patients were assessed for optimal veins in their upper extremities. Technically, the clinician has nine quadrants in the arm, cubital fossa, and forearm region created by three regions from left to right, i.e., the basilic, brachial, and cephalic veins. However, the forearm quadrant of the brachial vein does not exist because the vein is not formed at that level; hence, we had eight available quadrants (Figure 1).

After the ultrasound screening, the clinician prepared the tray and equipment. Quadrants and veins were selected based on vein size and clinician comfort. Based on the size of the vein, the clinician decided whether to place a 3 Fr or 4 Fr catheter (LeaderCath^TM^). Real-time ultrasonography was used to localize and guide the cannula. Sonography was performed with a 4-13 MHz frequency probe using a Logiq P9 ultrasound device (GE Healthcare, Anaheim, CA, USA). Using the linear probe, we performed gray-scale sonography to obtain basic anatomical information and capture the vein of choice in the out-of-plane technique. Color Doppler imaging was used to confirm the vessels. Vein diameter was measured during the pre-scan in an out-of-plane approach in B mode.

The vein diameter is divided by the outer diameter of the catheter to get the vein-catheter ratio (VCR). The outer diameter of a 3 Fr catheter is 0.9 mm, and for 4 Fr it is 1.2 mm. When there is difficulty puncturing the vein, the clinician removes (exits from the skin) and reinserts the needle at different points, which is considered a second attempt. The duration of the procedure was recorded using a timer. The duration from the placement of the probe in the sterile area until backflow from the catheter was confirmed, which included guidewire insertion and catheter placement. Pre-scan, preparation of the equipment tray, and local infiltration of lignocaine were not accounted for in the duration of the procedure in this study.

**Figure 2:**
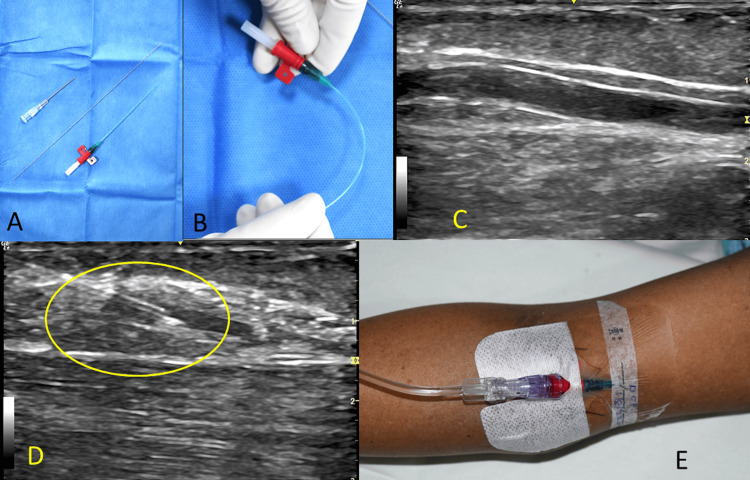
Summary of the procedure A: Procedure tray with the introducer needle, guidewire, and catheter; B: A catheter showing flexibility and anti-kinking nature; C: Vein of interest seen in the in-plane view; D: Needle entering vein (circled area); E: The catheter is secured

A qualified anesthesiologist (intensivist) with more than five years of experience performed all procedures. All had reasonable experience in placing PVC under sonography, but we did not attempt any training sessions concerning placement with LeaderCath^TM^. After briefly explaining the procedure and its risks and benefits, we prepared the patients. The patient lay supine with the arm abducted and externally rotated on a separate table. An alcohol-based cleansing solution was used for disinfection. After the screening ultrasound, the appropriate vessel was captured in an in-plane view, along the course of the vein (Figure 2 C). 

Then, 0.5 to 1 mL of 2% lignocaine was used to anesthetize the injection point. Following this, the access needle was introduced into the skin and subcutaneous tissue using the sonography-guided in-plane technique (Figure 2 D). While directly visualizing the course of the needle in the image, we punctured the vein. After confirming the backflow of blood, the guidewire was advanced. The placement was double-checked by sonographically confirming the guidewire inside the vessel in the out-of-plane view. The catheter was advanced over the guidewire, and the guidewire was removed. We again confirmed the position of the catheter by aspiration of blood through the catheter using a syringe.

The LPCs were secured with sutures (Figure 2 E), and no additional charges were applied to the patients for the LeaderCath^TM^ or ultrasound. All patients who had LPC, irrespective of their indication for insertion, underwent blood sampling through the LPC after discarding the initial 10 mL. After sampling, the lumen was flushed with saline in a prefilled 10 mL syringe, and medications, if on flow, were continued. At all points in the dwell time, we were cautious about the injection speed during flushing and avoided 2 mL syringes to prevent excessive pressure and catheter rupture. Noradrenaline or vasopressors were administered through the LPC. Failure of the LPC was defined as the absence of forward or backflow for four days [6].

The data collected included patient demographics, diagnosis, reason for LPC insertion, insertion site, catheter size, procedure duration, and number of attempts. The use of hyperosmolar drugs and sampling frequency were also recorded. Patients who had CVC and LPC at different periods of their ICU stay were asked to give a score of 10 on the comfort of the LeaderCath™ LPC and CVC, where 10 represents the maximum comfort level. The nursing staff were also asked the same questions. For any fever, we would send blood samples for microbiological culture, and the presence of bacterial growth in the blood was documented. Any unilateral upper limb edema, redness, induration, or pain led to the removal of the LPC.

Statistical analysis

All continuous variables are represented as medians with interquartile ranges and categorical variables as numbers and percentages. Continuous variables were compared using the Mann-Whitney U test, and the chi-squared test was used for categorical variables. We considered failure to use LPC for four days as LPC failure and obtained a receiver operating characteristic (ROC) curve for vein diameter and VCR to determine the optimal point at which LPC longevity was affected. A binary logistic regression analysis was performed to identify the risk factors associated with LPC failure. For all tests, the p-value of < 0.05 is considered statistically significant. All data were entered in Microsoft Excel (Microsoft Corp., Redmond, WA, USA), and statistical analyses were performed using SPSS Statistics for Windows version 28.0 (IBM Corp., Armonk, NY, USA).

## Results

A total of 38 patients with LPC secured in the deep arm veins were included in this descriptive study. The median age of the participants was 55 years (IQR: 44 to 70 years), with a predominance of male patients (84.2%) (Table 1).

**Table 1:** Patient and procedure characteristics for long peripheral catheter insertion into deep-arm veins All values are expressed as either median with IQR or number with percentage. LPC: Long peripheral catheters, CLD: Chronic liver disease, PVC: Peripheral vein catheter, CVC: Central venous catheter

Parameter		Number (percentage); Total n= 38
Age (in years)		55 (44, 70)
Gender		32 (84.2%)
Diagnosis	Decompensations in CLD	19 (50%)
	Acute liver failure	2 (5.3%)
	Pancreatitis	7 (18.4%)
	Post liver transplant	7 (18.4%)
	Others	3 (7.9%)
Indication for LPC	CVC to be removed and needs frequent sampling	12 (31.6%)
	PVC in situ and need of frequent sampling	6 (15.8%)
	Difficult PVC access	17 (44.7%)
	Others	3 (7.9%)
Site of insertion	Arm	15 (39.5%)
	Cubital fossa	23 (60.5%)
Vein of placement	Basilic	17 (44.7%)
	Brachial	18 (47.4%)
	Cephalic	3 (7.9%)
Catheter used	3 Fr	20 (52.6%)
	4 Fr	18 (47.4%)
Vein diameter (internal) in mm		3.4 (2.7, 4.3)
Vein catheter ratio		3.2 (2.6, 3.9)
Attempts taken to place the LPC	1	28 (73.7%)
	2	10 (26.3%)
Duration of procedure in seconds		180 (90, 300)
Arterial puncture		1 (2.6%)
Presence of backflow 24 hours from insertion		34 (89.5%)

The most common indication for LPC placement was difficult peripheral venous access (17 patients (44.7%)), followed by removal of an existing CVC in patients requiring frequent sampling (12 patients (31.6%)). The LPC was placed in the cubital fossa in 23 patients (60.5%) and the upper arm in 15 (39.5%). The brachial vein was the most commonly used vein for LPC placement, followed by the basilic vein; the cephalic vein was used in only three patients (7.9%).

**Figure 3:**
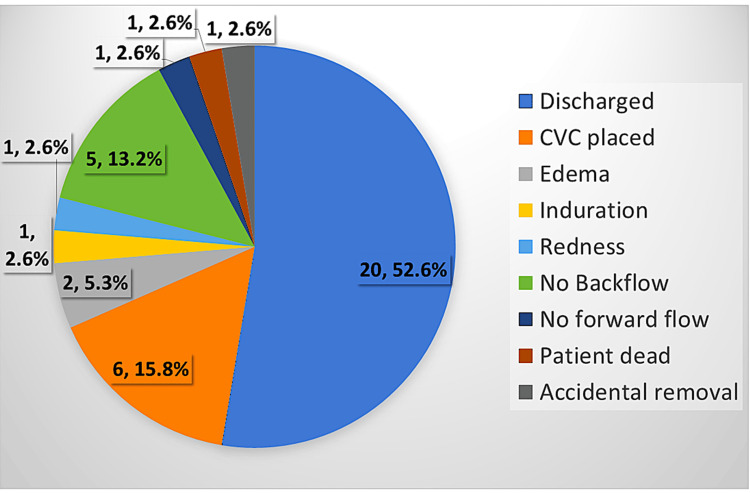
Pie chart showing the reasons for LPC removal All values are expressed as number with percentages. LPC: Long peripheral catheter, CVC: Central venous catheter

The median vein width in our study was 3.4 mm (IQR 2.7-4.3), and the median VCR was 3.2 (IQR 2.6-3.9). We used a 3 Fr catheter in 20 (52.6%) patients and a 4 Fr in 18 (47.4%) patients. The median duration of the procedure was three minutes (IQR 1.5-10 minutes). On 10 occasions (26.3%), the clinician required a second attempt to place the LPC. We did not face failure to place the LPC. On one occasion, the brachial artery was inadvertently punctured (2.6%). The patient was managed with compression for a few minutes, and there was no hematoma or continuous oozing. On four occasions (10.5%), backflow was not present on the same day of insertion (within 24 hours). In this situation, we obtained samples by phlebotomy each time, used the LPC for forward flow, and administered medications and fluids. In one case (2.6%), accidental removal occurred in a patient with encephalopathy who pulled out the LPC, resulting in its displacement on day three. One patient (2.6%) with pancreatitis did not have forward flow (within 48 hours). If there was no forward flow, the LPC was deemed unnecessary and removed, and a conventional PVC was placed for venous access. Among the five patients who did not have backflow within four days of insertion, three (60%) had pancreatitis when compared to the four patients (14.2%) among the 28 patients who had backflow beyond four days (χ2= 5.3, p= 0.02). One patient (2.6%) had edema surrounding the insertion site by 48 hours, which was the reason for the removal of the LPC; the edema resolved within a day after removal. The same patient also experienced a peri-catheter leak. Another patient with pancreatitis developed site induration within 48 hours (Figure 3).

**Figure 4:**
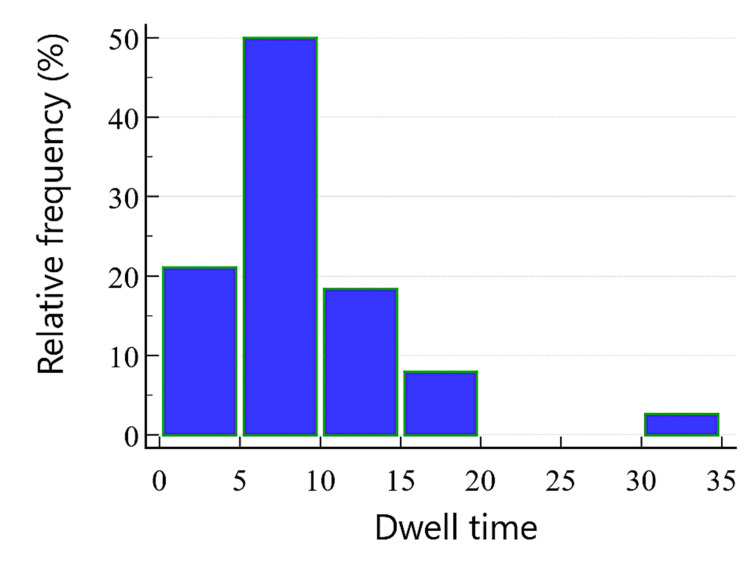
Histogram showing the distribution of dwell time for various LPCs The dwell time (x-axis) is expressed in days. LPCs: Long peripheral catheters

In total, 26 patients (68.4%) maintained effective backflow and forward flow throughout the dwell time, allowing for consistent blood sampling. Of these, 20 patients (52.6%) were discharged without requiring further catheterization, while six patients (15.8%) required CVC later in their ICU stay due to clinical deterioration. The median dwell time for LPCs was eight days (IQR five to 11 days; maximum 30 days) (Figure 4), with blood sampling required twice daily in 21 (55.3%) patients on the day of insertion. Blood sampling was required every sixth hour in 16 patients (42.1%) and the fourth hour in one patient (2.6%). Nine patients reported discomfort during the LPC dwell time (Table 2).

**Table 2:** Course and events during the dwell time of LPC All values are expressed as either median with IQR or number with percentage. LPC: Long peripheral catheter

Characteristics		Number (percentage), Total n= 38
Dwell time of LPC in days		8 (5, 11)
Sampling frequency on the day of insertion	12^th^ hourly	21 (55.3%)
	6^th^ hourly	16 (42.1%)
	4^th^ hourly	1 (2.6%)
Use of hyperosmolar drugs at any point of time in the dwell period		24 (63.2%)
Patients having discomfort with LPC at any point of time in the dwell period		9 (23.7%)
Fever at any point of time in the dwell period		3 (7.9%)
Microbiological growth in cultures (from the sample prior to LPC insertion)		3 (7.9%)
Microbiological growth in cultures (from the sample taken after LPC insertion)		1 (2.6%) *Candida auris*
Catheter that did not serve the purpose for 24 hours		4 (10.5%)
Catheter that did not serve the purpose for four days		10 (26.3%)

Fever occurred in three patients in the post-insertion period; one had a positive blood culture for *Enterococcus faecium* (obtained prior to LPC insertion), while two showed no bacterial growth. One patient with persistent leukocytosis was found to have a blood culture positive for *Candida auris*, which led to LPC removal on day 30 due to fungal growth; this patient had an ICU stay of approximately 80 days. Patient and nursing staff comfort levels with LPCs were assessed on a scale of 1-10, with 10 representing maximum comfort. Among the 12 patients who had both CVC and LPC during their ICU stay, comfort scores were comparable between devices from the care provider’s point of view; however, LPCs yielded higher comfort ratings from the patients (8 (6, 8) vs. 5 (5, 6); p = 0.008) (Table 3).

**Table 3:** Patient and nursing staff comfort levels with LPC and CVC A numerical rating scale was used to elicit the comfort level on a scale of 1 to 10, with 10 being very comfortable. The effect size was calculated from the z-score categorized as small effect, r<0.3; medium effect, r = 0.3 to 0.5; and large effect, r>0.5. LPC: Long peripheral catheter, CVC: Central venous catheter

Subgroup analysis	Number	Z-score	p-value
Patients’ comfort on a scale of 10 (10 being very comfortable) for LPC	8 (6, 8)	2.61	0.008
Patients’ comfort on a scale of 10 for CVC	5 (5, 6)		
Care provider’s comfort on a scale of 10 for LPC	8 (7, 9)	0.34	0.76
Care provider’s comfort on a scale of 10 for CVC	8 (7, 9)		

Excluding the patient who died within a few days and the accidental removal, we took 10 occasions in which we failed to utilize the LPC for more than four days as LPC failure and evaluated the associated factors. Although the sample size was small, we attempted to predict the factors associated with LPC failure using a binary logistic regression analysis. The only significant factor in the univariate analysis was the etiology of pancreatitis (OR = 21.3 (95% CI: 1.73, 263.7)). Owing to the small sample size, the 95% CI was unusually wide, and we did not perform multivariate analysis because only one factor was significant. None of the other factors were found to be significant. Thus, we inferred that the site of insertion, diameter of the vein, VCR, size of the LPC, and use of hyperosmolar drugs were not associated with LPC failure. However, a larger study would provide better insight.

Receiver operating characteristic curve analysis indicated that vein diameter ≤3.1 mm (AUC = 0.65, p = 0.18) and VCR ≤3.5 (AUC = 0.66, p = 0.15) were predictive of LPC failure within four days. Of the total 38 patients, 11 had both vein diameters ≤3.1 mm and VCR ≤3.5. Of these 11 patients, five (45.5%) experienced LPC failure (vs. five of the remaining 27 patients (18.5%); p = 0.08). Ultrasound assessment of 24 patients after LPC removal revealed thrombus formation in the catheterized vein in 15 patients (62.5%), with no evidence of thrombus in the axillary vein.

## Discussion

The LeaderCath™ is conventionally used for arterial cannulation and is recommended by the manufacturer. However, studies have used LeaderCath™ for venous access [7,8]. As a long peripheral catheter (LPC), ranging from 6 cm to 15 cm in length, it occupies an intermediate position between conventional PVCs and CVCs. The LPCs are inserted under real-time ultrasound guidance using the Seldinger technique and offer cost benefits compared to CVCs. The terminology differs and is variably mentioned in the literature. We used to call this a deep-arm vein catheter, whereas Qin et al. suggested adopting the name long peripheral catheters, which we think is rational [5]. The flexible body and anti-kink collar of the LeaderCath™ help maintain catheter patency, even in patients with significant peripheral edema, a common ICU issue due to fluid resuscitation and hypoalbuminemia [9,10].

Conventional PVCs have a high incidence of infiltration due to their shorter length and kinking risks, particularly in edematous patients [6,11,12]. Studies suggest that LPCs are better suited than PVCs for challenging venous access cases, particularly with ultrasound-guided in-plane insertion techniques, which require longer access needles and catheters. The LeaderCath™ catheter (8 cm for 3 Fr and 10 cm for 4 Fr) was positioned between the PVC and LPC in terms of cost and functionality.

Most of our patients had decompensations due to underlying chronic liver disease, and the next most common etiology of ICU admission was pancreatitis. Difficult venous access was the most common reason for LPC placement. Technically, whoever had LPC in situ had blood sampling performed from the LPC. We believe that it was too invasive to place an arterial catheter in a clinically improving patient. If not for LPC, we would have used needle phlebotomy to obtain samples. For most patients who recovered from a critical illness, we obtained samples twice a day until discharge or transfer to the ward. The cubicle fossa was the most comfortable site for insertion. The brachial and basilic veins are the most frequently used. The catheter size was selected based on an ultrasound assessment of vein size. The overall success rate of the LPC placement was good. A second attempt was needed in 10 cases (26.3%), and an inadvertent arterial puncture occurred in one case (2.6%). This is in agreement with the success rate of ultrasound-guided PVC placement in the literature of 91% and 99% [4,13]. The median duration of the procedure was three minutes. We encountered an obliteration of the vein after local anesthetic infiltration. On four occasions (10.5%), we failed to achieve good backflow on the day of insertion, and the incidence of LPC failure was higher in patients with pancreatitis. Although LPCs provide reliable access in most patients, a 31.6% failure rate within four days warrants further investigation to assess whether the comfort benefits outweigh the failure risk. Further studies directly comparing CVC and dialysis catheter (DC) along with infection rates and colonization rates might provide a better picture of whether it is worth taking the risk of 31.6% failure.

The failure rate might be high concerning the dwell time of ultrasound-guided PVC. Keyes et al. reported a failure rate of 8% within an hour of insertion and attributed it to the short length of the PVC [13]. In a study conducted by Dargin et al., a lower proportion of catheters (19%) survived for > 72 hours for PVC inserted under ultrasound guidance, whereas in our study, 68.4% of LPC survived at 96 hours [6]. In the same study, 47% of PVC failed within 24 hours, and the infiltration rate was 28%, which was too high when compared to LPC in our study. The same author concluded that ultrasound-guided PVC can be a good option for difficult venous access, but with expected brief use, like the administration of contrast in the radiology suite or similar situations in the emergency department, and does not recommend it for longer use [6]. Another study that used guidewire-based insertion of PVC under USG guidance demonstrated the incidence of infiltration, failure, and other complications at a rate similar to that in our study [4]. In a meta-analysis, the incidence of infiltration with LPC was 0.9%, which is much lower than that seen with PVC [14]. Even though we did not compare the ultrasound-guided PVC and LPC, the overall complication rate of PVC is said to range from 47% to 65% according to different studies [15]. Including redness, arterial puncture, edema, induration, and accidental removal as complications in our study would translate to 13.2% for LPC. However, a direct comparison between LPC and conventional PVC would have provided a better picture.

Limitations

The first limitation is the small sample size. Further studies with larger cohorts are needed to draw conclusions. The site of insertion, choice of catheter (3 Fr or 4 Fr), and vein of insertion were decided according to eye-balling and subjective decision, and the lack of an algorithm in the approach is a limitation. We also did not measure the tip position concerning the axillary vein. Another limitation is that we could only compare the comfort scores between the CVC and LPC groups and that too only in a subgroup of 12 patients. Therefore, this was not a direct comparison between CVC and LPC concerning procedure duration, complications, cost involved, and infection rate; further studies are needed to directly compare the two. We also did not perform a comparison with a group that underwent USG-guided conventional PVC placement. We did not send the tip of our LPC or the LPC for microbiological culture to study the colonization rate. Further studies are needed to compare LPC with peripherally inserted central catheters, CVC, and PVC. Another limitation is that we took an arbitrary value of four days to define LPC failure.

## Conclusions

Long peripheral catheters inserted under ultrasound guidance offer enhanced peripheral venous access, providing backflow channels for blood sampling and longer dwell times than conventional PVCs. In our study, the success rate was good, and the procedure was not time-consuming. Complications were fewer and better tolerated by patients. These advantages make LPC a valuable option for difficult venous access in ICU settings.
